# Stress Load and Ascending Aortic Aneurysms: An Observational, Longitudinal, Single-Center Study Using Computational Fluid Dynamics

**DOI:** 10.3390/bioengineering11030204

**Published:** 2024-02-22

**Authors:** Fabiula Schwartz de Azevedo, Gabriela de Castro Almeida, Bruno Alvares de Azevedo, Ivan Fernney Ibanez Aguilar, Bruno Nieckele Azevedo, Pedro Soares Teixeira, Gabriel Cordeiro Camargo, Marcelo Goulart Correia, Angela Ourivio Nieckele, Glaucia Maria Moraes Oliveira

**Affiliations:** 1 Department of Cardiology, Federal University of Rio de Janeiro, Rio de Janeiro 21941-913, RJ, Brazil; glauciamoraesoliveira@gmail.com; 2 Research and Teaching Department, Instituto Nacional de Cardiologia, Rio de Janeiro 22240-006, RJ, Brazil; gabccamargo@gmail.com (G.C.C.);; 3 Department of Mechanical Engineering, Pontifical Catholic University of Rio de Janeiro, Rio de Janeiro 22451-900, RJ, Brazil; gabrielacastroalmeida@gmail.com (G.d.C.A.); azevedo.b@gmail.com (B.A.d.A.); iibanez@puc-rio.br (I.F.I.A.); bnazevedo@puc-rio.br (B.N.A.); nieckele@puc-rio.br (A.O.N.); 4 Fit Center—Clínica de Performance Humana, Niterói 24220-331, RJ, Brazil; pedrosote@gmail.com

**Keywords:** ascending aortic aneurysm, computational fluid dynamics, wall shear stress, wall pressure

## Abstract

Ascending aortic aneurysm (AAoA) is a silent disease with high mortality; however, the factors associated with a worse prognosis are not completely understood. The objective of this observational, longitudinal, single-center study was to identify the hemodynamic patterns and their influence on AAoA growth using computational fluid dynamics (CFD), focusing on the effects of geometrical variations on aortic hemodynamics. Personalized anatomic models were obtained from angiotomography scans of 30 patients in two different years (with intervals of one to three years between them), of which 16 (53%) showed aneurysm growth (defined as an increase in the ascending aorta volume by 5% or more). Numerically determined velocity and pressure fields were compared with the outcome of aneurysm growth. Through a statistical analysis, hemodynamic characteristics were found to be associated with aneurysm growth: average and maximum high pressure (superior to 100 Pa); average and maximum high wall shear stress (superior to 7 Pa) combined with high pressure (>100 Pa); and stress load over time (maximum pressure multiplied by the time interval between the exams). This study provides insights into a worse prognosis of this serious disease and may collaborate for the expansion of knowledge about mechanobiology in the progression of AAoA.

## 1. Introduction

Cardiovascular diseases are the leading cause of death worldwide. Among them is ascending aortic aneurysm (AAoA), which is a severe disease characterized by silent progression and high mortality due to complications such as rupture or dissection of the aorta [1]. AAoA is a dilatation of the initial segment of the aorta, the largest human body artery, which receives all the blood flow pumped by the left ventricle [2]. Asymptomatic, its diagnosis often occurs as an acute aortic syndrome (a life-threatening condition with aortic wall rupture), via a postmortem diagnosis or as an incidental finding when a patient undergoes imaging tests due to other causes [3]. Surgical correction of the aneurysmal aorta alters the natural course of the disease, preventing complications and reducing mortality [1,2].

Aneurysm growth, as defined by current guidelines [2,3], is based on the aortic diameter and usually occurs at a rate of 0.1 cm/year among degenerative aneurysms, which make up the most common form of AAoA. Degenerative aneurysms have decreased connective and muscle tissue on the arterial wall, favoring their dilation and rupture. Surgery is planned based on the diameter of the ascending aorta, obtained by imaging, under periodic monitoring [1,2,3]. In this context, in recent years, specific geometry studies have emerged to assess the risk of various cardiovascular diseases [4,5,6]. However, comparisons of aortic diameter are hampered by the asymmetry of this artery [7], and it has been shown to be an insufficient risk predictor in patients with AAoA [8]. In addition to the increase in the diameter of the aorta, susceptibility to complications occurs due to hemodynamic stress caused by hypertension, connective tissue diseases, and atherosclerosis, for example [1,2]. Thus, exploration of other comprehensive methods for assessing aneurysm growth is mandatory. Den Hartog et al. [9] demonstrated high reproducibility of aortic volume measurements via magnetic resonance imaging. Renapurkar et al. [10] showed an increase in the volume of abdominal aortic aneurysms by computed tomographic angiography (CTA) without a corresponding increase in the maximum aortic diameter. According to Raghavan et al. [11], the volume rather than the diameter of abdominal aortic aneurysms is a better indicator of high wall shear stress (WSS). Meyrignac et al. [12] also showed that combined analysis of lumen volume and WSS was associated with abdominal aortic aneurysm growth. Recently, Xiao et al. [13] studied, by fluid–structure interaction (FSI), one hundred reconstructed AAoA geometries and the relationship between hemodynamic conditions, ascending aortic volume, ascending aortic curvature and aortic relations measured from reconstructed three-dimensional (3D) models. The authors described a strong link between increased volume of the ascending aorta and critical hemodynamic conditions. Xiao et al. [13] highlighted that volumetric measurements make it possible to verify the increase in the entire ascending aorta rather than just a section of it, concluding that volumetric measurements are a promising tool for the clinical management and prevention of problems in patients with AAoA.

The differences in aneurysm growth behaviors among patients and the mechanisms associated with a worse prognosis are not completely understood [14]. The condition of out-of-control risk factors such as hypertension or smoking is known to be related to the progression of an aneurysm [15], and it is well known that the control of risk factors is not enough to contain aneurysm growth; thus, it is mandatory to identify other conditions that could influence the aneurysm progression rate.

In the last decade, some review articles have discussed the use of computational fluid dynamics (CFD) to enhance the understanding of hemodynamic patterns in cardiovascular disease [16,17]. In this context, the employment of CFD to investigate aortic diseases [18,19,20,21,22,23,24], particularly in aneurysmatic ascending aortas [25,26], has been the subject of numerous studies. Current evidence shows that employing CFD for flow analysis in healthy or aneurysmal aortas can contribute to understanding disease etiology by providing insights into the fields of velocity, shear stress, vorticity and identification of recirculation regions [22].

Simão et al. [25] studied blood flow using CFD in two patients with AAoA and one normal control case. The authors defined “high shear stress” as values exceeding the average value found in the control case (5 Pa). They described a reduction in shear stress in the dilated aortic area, in addition to the formation of vortices and locally reversed flow, related to a possible association with low flow velocity and variation in shear stress. In this study, the streamlines in the normal aorta did not form vortices, but these formations were observed in cases with aneurysms. Salmasi et al. [27] evaluated WSS using CFD in 11 of 33 patients with AAoA who had undergone four-dimensional cardiovascular magnetic resonance flow imaging (4D-flow CMR). The authors found associations between greater left ventricular outflow tract aortic angles and larger aneurysm diameters, greater velocities, and increased WSS at the aortic curve. A combination of these flow patterns in the region could be associated with a worse prognosis for the aneurysm. Salmasi et al. [28] reinforced the postulate that the aneurysm is a “flow-mediated” disease. Boczar et al. [29] prospectively studied individuals with AAoA to determine the role of aortic stiffness and pulsatile arterial load on future aneurysm growth. The authors concluded that lower arterial compliance and measures of pulsatile hemodynamics were independently associated with future aneurysm expansion. A study by Zhu et al. [30] evaluated the flow in surgically corrected aortas and correlated certain flow patterns with dilation of the residual dissected aorta.

Contemporaneous knowledge lacks a study with a large number of patients in which CFD is applied to analyze the risk of AAoA growth, comparing groups of patients over time with and without aneurysm growth. The aim of the present study was to numerically determine blood flow in two groups of patients, one presenting AAoA growth and the other not, focusing on the effects of geometrical variations on aortic hemodynamics, identifying the relationship between the distribution of wall pressure and wall shear stress with AAoA growth through a statistic analysis, including a novel parameter of the stress load over time (i.e., the expression of time in an annualized rate analysis).

## 2. Materials and Methods

The methodology of this study had three fronts. The first consisted of selecting patients with AAoA. The second corresponded to the numerical simulation of blood flow along the ascending aorta. Finally, a statistical analysis of the data obtained was carried out to determine the main characteristics of the groups with and without aneurysm growth.

### 2.1. Patient Selection

**Figure 1:**
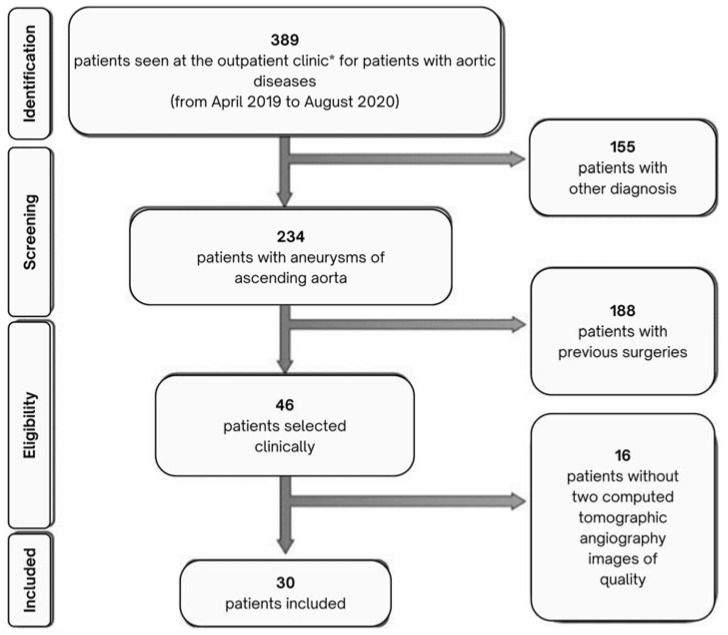
Selection of patients with ascending aortic aneurysms for this longitudinal study using computational fluid dynamics. (*) National Institute of Cardiology, Rio de Janeiro, RJ.

The study included a convenience sample of patients with AAoA who were examined at an outpatient clinic specializing in aortic diseases (Instituto Nacional de Cardiologia, Rio de Janeiro, RJ, Brazil) between April 2019 and August 2020. We excluded patients with a history of cardiac surgery, percutaneous intervention of the aortic valve or ascending aorta, aortic coarctation, ascending aortic dissection, collagen disease or Marfan syndrome. Patients with unavailable CTA images or inadequate radiological techniques (e.g., artefacts or no use of contrast agent) were also excluded. All CTA exams were performed as recommended by the attending medical team. The flowchart in Figure 1 shows the procedure used to select 30 patients among 389 patients with AAoA.

### 2.2. Data Collection and Image Acquisition

Personalized anatomic geometries were obtained using CTA images of the aorta at two different time points, with an interval of 1 to 3 years between each exam. The CTA images (in DICOM format) were acquired with intravenous iodinated contrast using a 64-slice computed tomography scanner (SOMATOM Sensation 64, Siemens, Forchheim, Germany). The valve effective orifice was also measured from the images, and a computational domain was subsequently constructed.

### 2.3. Geometry Segmentation and Three-Dimensional Computational Model

**Figure 2:**
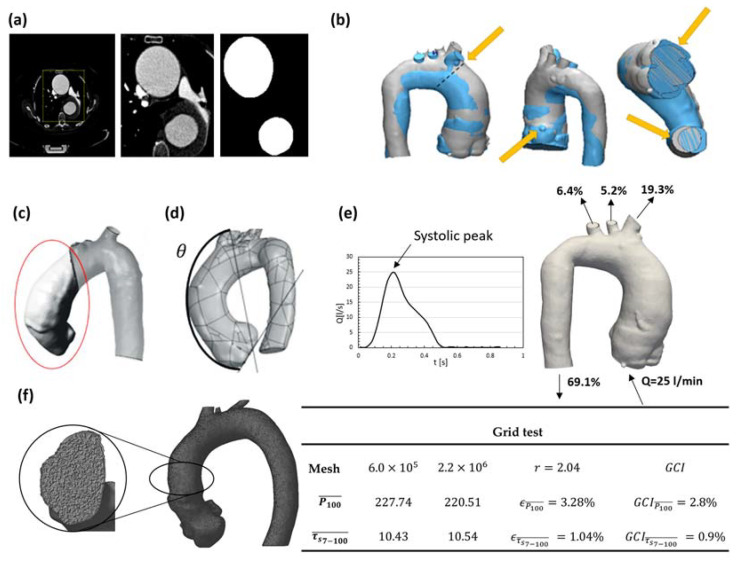
Workflow to generate the computational domain: (**a**) segmentation of angiotomography images of the thoracic aorta; (**b**) comparison criteria between two aortas of the same patient in different years (blue and gray) by superposition, in which the yellow arrows indicate the anchoring points (superposition of the brachiocephalic trunks and the right coronary arteries), and the aortic valve and descending aorta sectioned; (**c**) definition of the region of interest of the ascending aorta; (**d**) definition of θ angle; (**e**) typical physiological boundary conditions: flow rate at the systole peak of the cardiac cycle, equal to 25 L/min, and percentage of outflow rate at each outlet; and (**f**) mesh and grid test data. $\epsilon =\left|\left(\phi_{i}-\phi_{i+1}\right)/\phi_{i+1}\right|;GCI=F_{s}\epsilon /\left(r^{p}-1\right),\;\phi =P\;or\;WSS$.

All the images were pre-processed using FIJI (Fiji is Just ImageJ 1.53c) [31]. After the DICOM images were transferred to FIJI, a large area that contained the aorta was demarcated. After that, only this area was considered, and a series of preprogrammed filters were used to ensure that all slices of the exam contained only aortic pixels (Figure 2a). Pixel size and slice thickness data were used for the reconstruction of the 3D model of the aorta to its actual size. As a final procedure to create the computational domain, allowing a comparison of the results of two exams of the same patient, a geometry overlapping was employed [26] (Figure 2b). This ensured the consistent positioning of the inlet valve, provided a common coordinate system, and allowed the definition of the same region of interest (Figure 2c) to compare their volumes. Due to the high number of patients and the lack of detailed images of each patient’s valves, the entry was considered as a circular orifice, adopting the effective diameter of the valve for each patient, as utilized by Al-Jumaly [24].

### 2.4. Patient Classification

The presence of aneurysm growth was determined by comparing the volume of the region of interest (highlighted in red; Figure 2c) using the 3D computational geometry of the first and second CTA images of each patient (Figure 2b). The region of interest was limited by the aortic annulus plane (inflow plane) and the plane passing by the brachiocephalic trunk outlet, indicated by θ. The angle θ was defined as the distance between the aortic annulus plane and the line connecting the brachiocephalic trunk centroid with the left main coronary artery centroid (Figure 2d). Further details can be found in Almeida et al. [26].

As discussed in the literature [10,11,12,13], the volume is more sensitive than the maximum diameter and can capture any change in the entire geometry. Thus, here, the volumetric measurement was employed to identify aneurysm growth since it allowed better methodological reproducibility than a localized measurement (impinging jet point) due to the anatomical variations of each patient. Growth was considered to have occurred when the volume increased by at least 5% between the exams [26]. The patients were then classified into two groups based on whether the aneurysm grew.

### 2.5. Flow Modeling

To determine blood flow, a constitutive model of blood rheology is necessary. In the present study, blood was modeled as a Newtonian fluid (dynamic viscosity μ = 3.5 cP [26,32]) since inside vessels with a large diameter such as the aorta, during the systolic peak, the deformation rate is higher than 50 $s^{-1}$, and the blood behaves like a Newtonian fluid [32,33,34]. A comparison of the flow prediction at the systolic peak inside aortas with aneurysms, employing Newtonian and non-Newtonian viscosity models [35], revealed similar flow patterns and wall tension distributions, corroborating the small impact of viscoelasticity on these large vessels. Further, the blood is considered incompressible [34], with the density set to ρ = 1054 kg/$m^{3}$.

Although the physiological flow is pulsatile, as a first approximation, it can be represented by a succession of stationary states. This is a convenient approximation since it substantially reduces the computing effort while maintaining the major flow characteristics [32,33,34,35]. Furthermore, as shown in Perocco [36], the critical condition with respect to high pressure and high WSS actually occurs at the systolic peak, indicating that this is a good approach for examining the worst scenario. Therefore, the flow was modeled in a steady state [37,38,39,40] considering the critical condition, i.e., with a flow rate of $Q_{in}$ = 25 L/min [41] at the valve inlet, corresponding to the peak of the systolic phase of the cycle (Figure 2e).

The maximum and minimal Reynolds number, based on the inlet velocity, and effective valve diameter for the group without aneurysm growth were equal to 8374 and 5280, and for the other group (with growth), they were 7368 and 4953, characterizing the inlet flow as turbulent. Turbulence [42] was modeled using the two-equation κ-ω SST model [43] as recommended by Celis [44], who carried out a numerical simulation of an aorta model and compared the prediction with the experimental data of de Azevedo et al. [45]. Gravity effects were neglected since pressure variations are dominant.

The mass and linear momentum conservation equations based on the RANS methodology that govern the blood flow field can be expressed as

(1) $$div\;V=0;div\left(\rho \;V\;V\right)=-grad\hat{p}+div\left[\left(\mu +\mu_{t}\right)\left(grad\;V+grad^{T}\;V\right)\right]$$

where ρ is the blood density, $\hat{p}=P+2/3\rho \kappa$, is a modified pressure, P is the pressure, κ is the turbulent kinetic energy, μ is the molecular viscosity, and **V** is the velocity vector. $\mu_{t}$ is the turbulent viscosity, defined according to the κ−ω SST model [24], as a function of the turbulent kinetic energy κ and ω, which is the specific rate of dissipation, requiring the solution of their conservation equations. This model is obtained by combining the κ−ε model with the κ−ω model through the blending parameter ξ, to take advantage of accurate formulations of the κ−ω model in the near-wall region and of the κ−ε model in the far field. The turbulent eddy viscosity is given by

(2) $$\mu_{t}=\frac{\rho \kappa }{\omega }\xi$$

where the turbulent kinetic energy κ and its specific dissipation ω are determined by the solution of their conservation equations.

Aiming to numerically evaluate the influence of aortic anatomy on the hemodynamic patterns, and following the approach of Xiao et al. [13], the same boundary conditions were applied for all patients, which are based on the physiological conditions described in the literature [41,42,43]. This approximation was made due to a lack of detailed information at each boundary for each patient, and it is also supported by the research of Madhavan and Kemmerling [46], who investigated the impact of inlet and outlet boundary conditions in CFD modeling of aortic flow using a geometry constructed from images. The authors reported flow variation only in the region very close to the valve. They also observed negligible differences in flow when testing different outlet conditions. In line with the current investigation, the same stroke volume and cardiac output for all patients was assumed. Consequently, the uniform boundary conditions served as control variables, allowing for the evaluation of the influence of ascending aorta anatomy without introducing bias.

Here, for all cases, a uniform velocity profile was imposed at the inlet, with 5% turbulence intensity and a characteristic length equal to the inlet effective diameter of each patient, with the same ventricular pressure. At each outflow, diffusion flux was neglected and the flow rate percentage distribution, based on a typical physiological condition as proposed by Alastruey et al. [47] was prescribed: descending aorta, 69.1%; brachiocephalic trunk, 19.3%; left carotid artery, 5.2%; and left subclavian artery, 6.4% (Figure 2e). A no-slip condition was imposed at the aortic wall, which was considered rigid, due to the small compliance that exists when its maximum diameter is reached during the systolic peak [32,48].

### 2.6. Numerical Modeling

To define the mesh for the built 3D geometry, a mesh independence test was carried out to guarantee small variations in the average pressure in the region of high pressure (P > 100 Pa), $\bar{P_{100}}$, and in the average WSS in the region of intersection of high shear stress ($\tau_{s}$ > 7 Pa) and high pressure (P > 100 Pa), $\bar{{\tau_{s}}_{7-100}}$. A mesh with approximately 2 × 10^6^ nodes (Figure 2f) with tetrahedral elements was defined for all the cases studied using the software ANSYS Meshing v2020 [49], which corresponded to a grid convergence index (GCI) [50] less than 2.8% (Figure 2f). The GCI of pressure and WSS were determined in relation to the finest mesh, with a safety factor $F_{s}$ equal to 2, when the mesh was refined by a factor of $r=h_{i}/h_{i+1}$ = 2.04, where $h_{i}$ is the average spacing. Furthermore, the maximum dimensionless wall distance ($y^{+}=\rho \;y\sqrt{\tau_{s}/\rho }/\mu$, where y is the distance of the first internal node to the wall, $\tau_{s}$ is the WSS, and ρ and μ are the density and molecular viscosity, respectively) was less than 4, with an average mesh size equal to 0.5 mm.

The Reynolds average conservation equations of mass, linear momentum, turbulent kinetic energy, and its specific dissipation were numerically solved by the finite volume method using ANSYS Fluent v2020 R1 [49] software. The equations were discretized using the second-order upwind scheme. The pressure–velocity coupling was solved with the SIMPLEC algorithm. The solution was considered converged when the residual of all equations was below $10^{-6}$.

### 2.7. Postprocessing of the Data

The flow field was determined for all patients, and the simulation results were postprocessed using the ANSYS CFD-Post tool [49]. Since the flow was modeled as incompressible, the pressure level is not needed for the solution, and all pressure results shown here are relative pressure in relation to the inlet ventricular pressure, which as informed, was considered the same for all patients.

To correlate the data between the two groups of patients (with and without aneurysm growth), we determined the mean and maximum relative pressure values in the area with pressure equal to or above the critical value of 100 Pa. This is a conservative threshold, selected based on the study of a healthy patient by Ibanez et al. [32], who found a pressure difference between the aortic valve and the ascending aortic wall of approximately 1 mmHg (133 Pa). According to Simão et al. [25], the critical WSS is 5 Pa, while Etli et al. [51] consider 9 Pa to be the critical WSS. Here, we considered 5 Pa and 7 Pa (the average of the values reported in both references). The mean and maximum pressure and WSS values in the intersection areas associated with both critical values were also determined.

### 2.8. Statistical Analysis

A statistical analysis [52] of the numerically obtained results was performed based on the data corresponding to the first CTA image. We also collected blood pressure, heart rate, serum creatinine, and echocardiographic data from medical records, all registered no later than 3 months from the selected CTA images.

Continuous variables with a normal distribution are presented as the mean ± standard deviation, while those without normal distribution are shown as the median and interquartile range [IQR]. Categorical variables are presented as frequencies (numbers and percentages) and confidence intervals. The analyzed variables were compared with the outcome of aneurysm growth using the chi-square test and Fisher’s exact test. The Shapiro–Wilk test was applied to verify the type of distribution of each variable. We used paired Student’s *t* tests to analyze variables with a normal distribution and the Mann–Whitney test for those with a non-normal distribution. We also considered the annualized rate of each independent predictor of aneurysm growth by a univariate logistic regression model, multiplying them by time (in years) between the first and the second CTA exams. Furthermore, a multivariate logistic regression analysis with stepwise selection of variables was also applied. Spearman’s rank correlation was employed to measure the strength and direction of the association between any two ranked variables [53]. The data were analyzed using Jamovi 2.2.5, R 4.0.2, and R Commander 2.7-2. All tests were two-tailed. An alpha error of 5% was accepted, and *p* values ≤ 0.05 were considered significant.

## 3. Results and Discussion

The baseline clinical and echocardiographic characteristics of the overall study population are shown in Table 1.

The population of the present study had conditions of risk for AAoA, including male sex, age, hypertension, dyslipidemia, smoking, and atherosclerotic disease. As recommended by the guidelines [2,3], blood pressure levels and heart rate were under control in our patient population. Nevertheless, approximately half of the patients experienced aneurysm growth (16, 53.3%), which had no significant correlation between clinical or echocardiographic variables and the aneurysm growth (*p* > 0.05, where *p* is the probability of Type I error). Data on the New York Heart Association functional class were obtained from 21 patients; among these patients, 15 (71.4%) had functional class I [54], including 10 (47.6%) in the group without aneurysm growth. Patients were maintained on medication to control risk factors such as hypertension, diabetes, and dyslipidemia during the period between CT angiogram scans. The time interval between the first and second CTA exams was not significantly different between the groups (*p* = 0.075).Table 2 shows the volume and the maximum diameter of the ascending aorta on the first and second CTAs and the percentile volume variation and diameter variation between both exams. The diameter and volume variations demonstrated a moderate positive correlation (0.402—Spearman rank correlation test).

**Figure 3:**
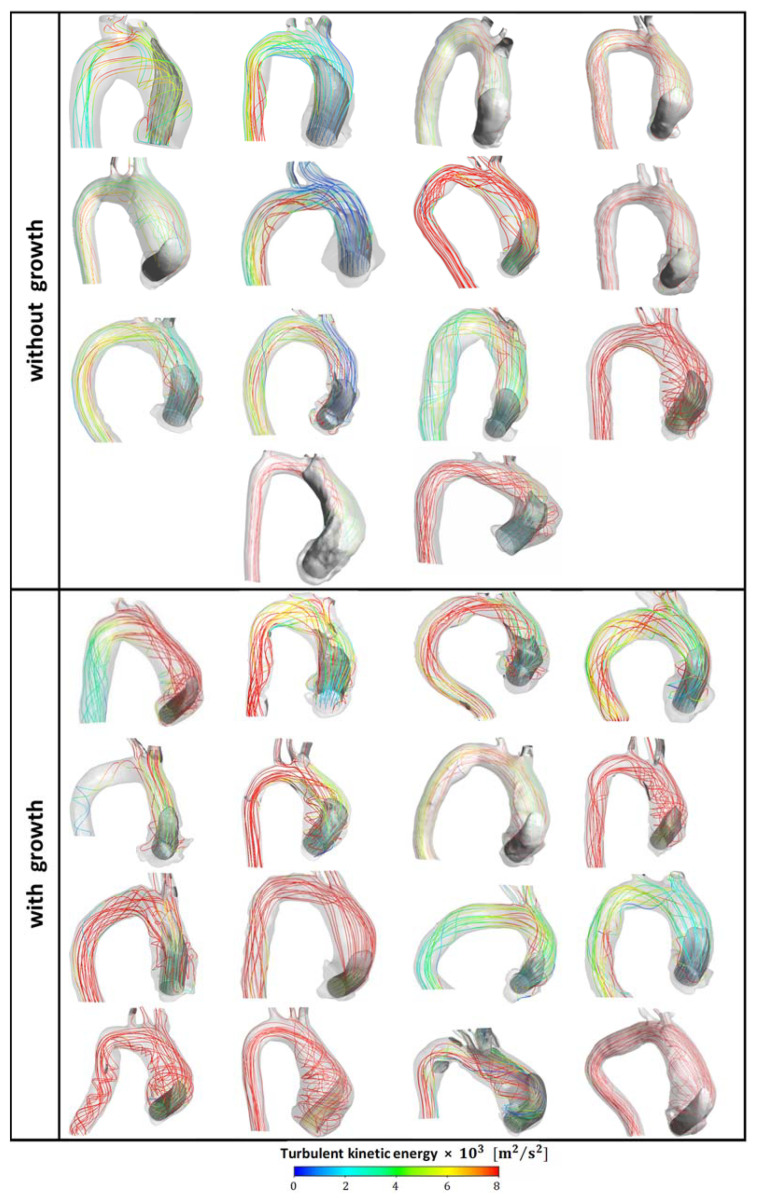
Patients with and without ascending aortic aneurysm growth. Streamlines colored according to turbulent kinetic energy with iso-surface at 50% of inlet velocity (first CTA).

To visualize the hemodynamic flow pattern of all patients, corresponding to the first CTA, Figure 3 illustrates the streamlines colored by the turbulent kinetic energy, with an axial velocity iso-surface (gray) equal to 50% of the inlet velocity, allowing the identification of the inlet jet, which is directed toward the anterior aortic wall. Due to the production of turbulent kinetic energy κ, the maximum κ inside the domain was 16 times higher than the inlet $\kappa_{in}$ for the aneurysm growth group and 9 $k_{in}$ for the non-aneurysm growth group. After the jet impinges the aortic wall, the stream is redirected and flows along the aortic arch. Flow recirculation around the main jet was observed, with high values of turbulent kinetic energy decaying along the aorta. Although qualitatively, the flow is similar for all the cases, and the intensity of the impinging jet leads to stronger recirculation and higher wall pressure and WSS.

**Figure 4:**
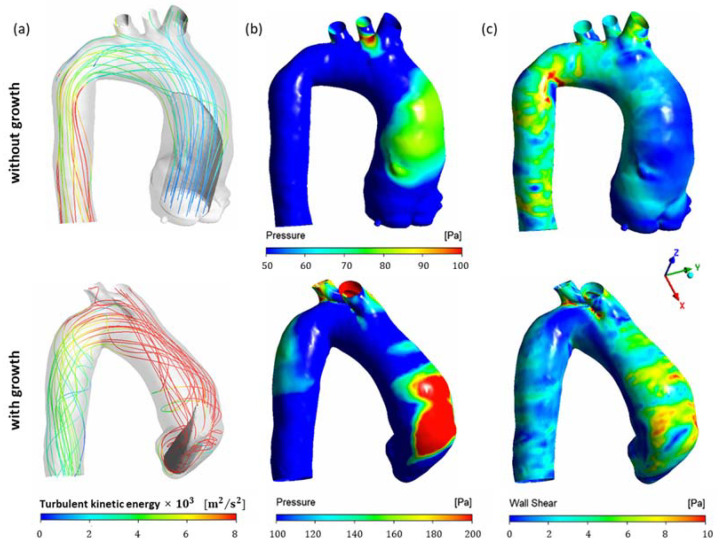
First CTA of patients with and without ascending aortic aneurysm growth: (**a**) Streamlines colored by turbulent kinetic energy with iso-surface at 50% of inlet velocity. (**b**) Pressure. (**c**) Wall Shear Stress.

To better highlight the flow differences between the two groups, Figure 4 shows the streamlines, wall pressure and wall shear stress of two specific patients without and with aneurysm growth. Figure 4a shows the streamlines colored according to the turbulent kinetic energy, with an iso-surface of iso-velocity, as presented in Figure 3. Note that the streamlines are aligned along the aorta for the patient without growth, while severe recirculation around the inlet jet can be observed for the patient with growth. Figure 4b,c illustrate the pressure difference between the wall and the inlet ventricular pressure, and WSS at the aorta. Patients with aneurysm growth presented higher wall pressure and WSS in the region of impingement of the inlet jet than patients without growth.

Table 3 presents the numerically predicted data corresponding to blood flow and the univariate correlation, showing that the maximum ($P_{max}$) and average pressure in the region of high pressure (P > 100 Pa, $\bar{P_{100}})$ are linked to aneurysm growth. High shear stress ($\tau_{s}$ > 7 Pa) is also linked to aneurysm growth, but only when combined with high pressure (≥ 100 Pa), $\bar{{\tau_{s}}_{7-100}}$. The authors believe that the no significance of wall shear stress alone is due to the sample size and follow-up time. A larger sample with a longer follow-up time will likely demonstrate significant differences in both pressure and wall shear stress, revealing the causal relationship between the stress load and the aortic remodeling process.

In the literature, wall pressure has been associated with vascular complications, including the progression of dilation and rupture of the thoracic aorta. Etli et al. [51] studied CFD hemodynamics in CTA images of two patients with AAoA and one normal control, evaluating the flow throughout the cardiac cycle. The systolic peak is the moment at which the greatest impingement force occurs on the vessel wall during the cardiac cycle. As in the present study, the flow patterns found by Etli et al. [51] at the systolic peak showed higher wall shear stress (reaching 9 Pa in the control case and 27 Pa and 39 Pa in the aneurysm cases) and higher wall pressure (18.56% and 23.8% higher, respectively, in patients with aneurysms). These results suggest that a region of the aortic wall subjected to a high stress load could be prone to vascular remodeling, thus increasing the volume of an aneurysm. The same study showed that patients with aneurysms had greater variation in shear stress on the vessel wall than control cases. Using a methodology similar to that used in the present study, Almeida et al. [26] studied nine cases of AAoA. The authors described complex vortices associated with an increase in mean pressure in the aortic wall (+13%) in five cases and a decrease in mean pressure (−18%) in relation to the baseline assessment in the remaining cases.

We also investigated possible correlations between groups regarding maximum turbulent kinetic energy, for which we observed median values of 0.08 [IQR 0.06–0.09] $m^{2}/s^{2}$ among the 14 patients without aneurysm growth and 0.07 [IQR 0.05–0.11] $m^{2}/s^{2}$ among the 16 patients with aneurysm growth, yielding a *p* value of 1 (no correlation).

A tangential force exerted on the vascular endothelium (or shear stress on the aortic wall) can alter the mechanobiology of the extracellular matrix and is closely related to aortic physiopathology [51,55,56,57]. The finding in the present study of an association between the area of intersection of high stress and high pressure and aneurysm growth suggests that superimposed mechanical forces on the aneurysm wall contribute to the risk of aneurysm progression. Several studies in the literature have evaluated the relationship between shear stress and aneurysms [58]. Petuchova and Maknickas [59] numerically evaluated AAoA considering aortic elasticity and oscillation during the cardiac cycle. The authors showed that the peak systolic velocity was lower in the aorta that had an aneurysm than in the normal vessel (1.18 m/s versus 1.9 m/s, respectively). Furthermore, the WSS range was greater in aortas with aneurysms than in healthy aortas (0–1 Pa versus 0.3–0.6 Pa, respectively). The authors also reported no significant difference in pressure between the two cases analyzed. In contrast, in the present study, we found that elevated pressure (≥100 Pa) correlated independently with aneurysm growth. As discussed by Sadeghi et al. [6], although the WSS has been recognized to influence the progression of vascular diseases, including AAoA progression and rupture, it is not yet well established in clinical practice as a tool for understanding prognosis or planning interventions.

Table 4 shows the statistical analysis of the annualized rate of numerical variables associated with aneurysm growth. Analysis of the burden of a risk factor over time can contribute to understanding the evolution of the aneurysm. In this approach, the independent significant variables obtained from the first CTA were multiplied by the time (in years) between the first and the second CTA. Statistically, an association was found between pressure load over time and the outcome of aneurysm growth. We observed that the variable ‘maximum pressure in the high-pressure area (i.e., area with pressure ≥ 100 Pa)’ during the time interval was associated with aneurysm growth according to a univariate logistic regression model, with a significant annualized rate of *p* = 0.0421. These findings indicated that the stress load may contribute to the mechanism of aneurysm expansion over time.

It is important to emphasizes that the current study was designed as an observational, longitudinal, and single-center CFD study. This research was retrospective and involved a non-probabilistic sample. Further, as listed by the modeling hypothesis, the present work presents some limitations, like applying identical boundary conditions for all patients and assuming a steady flow, i.e., ignoring pulsatility. Therefore, the findings cannot be generalized to all patients with AAoA. Although the impact of the patients’ anatomy in the flow field was examined and correlated with aneurysmal aortic growth, the valve shape coupled with the number of aortic valve cusps was not addressed. The presence of eccentric aortic flow has been demonstrated in patients with bicuspid aortic valve flow [48], and this flow may be associated with an increased risk of AAoA; this topic should be investigated in future work. As mentioned above, we presented an anatomy-specific study focusing on the critical condition of the maximum flow rate. Furthermore, the same boundary conditions were applied for the whole cohort, since detailed information of each patient was not available. Nevertheless, significance among the variables was obtained, highlighting which hemodynamic variables are more crucial to be monitored due to their influence on AAoA. A numerical study of the AAoA flow throughout the cardiac cycle could reinforce the conclusions obtained at the systolic peak in the present study.

As future perspectives, extending the analysis to encompass the entire cardiac cycle could expand the understanding related to the influence of hemodynamics on the growth of AAoA. A patient-specific simulation investigation, incorporating different boundary conditions, can also deepen the insights into this matter. Furthermore, a study involving a larger population employing machine learning techniques [60,61] can facilitate the exploration of the hypotheses generated in the present study.

Personalized CFD knowledge can clarify hemodynamic metrics and elucidate the prognostic impact of flow patterns, influencing aortic disease management and intervention recommendations to potentially save lives [4,61]. The development of software integration could automate the process, making it more cost-effective and efficient, thereby facilitating the expansion of aortic flow study using CFD and its application as a clinical tool.

## 4. Conclusions

In the present work, thirty patients with AAoA were selected and subdivided into two groups: with and without aneurysm growth throughout time. A comparative analysis between the two groups of the flow distribution, wall pressure, and WSS was performed. It was shown that statistical differences associated with AAoA growth obtained were: high wall pressure (P ≥ 100 Pa), high WSS ($\tau_{s}\geq$ 7 Pa), and high pressure and stress load over time (maximum pressure in the high-pressure area multiplied by the time interval between the exams). This perspective contributes to the potential expansion of knowledge about mechanobiology in the progression of aortic vascular diseases.

The present study showed that CFD, a noninvasive technique, applied to CTA scans performed regularly in the clinical monitoring of patients with AAoA, can contribute to a better understanding of the risk of AAoA progression by allowing us to numerically analyze hemodynamic behavior through specific aortic geometries. These findings may support further investigations of flow behavior considering physiological and patient-specific conditions, contributing to more individualized management in the future, and enabling optimization of the surgical moment for patients with this serious disease.

## Figures and Tables

**Table 1:** Baseline profile and statistical analysis of clinical and echocardiographic variables independently associated with AAoA growth.

Variables			Total (*n*)	Values	Growth		*p* Value
					No	Yes	
Clinical data	Sex	Male—*n* (%)	20	20 (66.7)	10 (33.3)	10 (33.3)	0.709
		Female—*n* (%)	10	10 (33.3)	4 (13.3)	6 (20)	
	Age (years)—mean ± SD		30	64.5 ± 10.6	62.9 ± 12.3 (*n* = 14)	65.9 ± 9 (*n* = 16)	0.453
	Interval between CTAs (years)—mean ± SD		30	1.9 ± 0.7	1.6 ± 0.7 (*n* = 13)	2.1 ± 0.7 (*n* = 16)	0.127
	Hypertension—*n* (%)		28	24 (85.7)	12 (42.9)	12 (42.9)	1.000
	Diabetes mellitus—*n* (%)		28	7 (25)	2 (7.1)	5 (17.9)	0.385
	Dyslipidemia—*n* (%)		27	16 (59.3)	8 (29.6)	8 (29.6)	1.000
	Chronic renal failure—*n* (%)		28	4 (14.3)	1 (3.6)	3 (10.7)	2.596
	Atrial fibrillation—*n* (%)		28	6 (21.4)	2 (7.1)	4 (14.3)	0.648
	Current or previous smoking—*n* (%)		26	12 (46.1)	5 (19.2)	7 (26.9)	0.695
	COPD—*n* (%)		25	2 (8)	0 (0)	2 (8)	0.480
	Obstructive CAD—*n* (%)		27	6 (22.2)	3 (11.1)	3 (11.1)	1.000
	Previous myocardial infarction—*n* (%)		27	3 (11.1)	2 (7.4)	1 (3.7)	1.000
	Stroke—*n* (%)		28	2 (7.1)	1 (3.6)	1 (3.6)	1.000
	Heart disease	Ischemic—*n* (%)	28	5 (17.9)	3 (10.7)	2 (7.1)	0.819
		Valvar—*n* (%)		5 (17.9)	2 (7.1)	3 (10.7)	
	SBP (mmHg)—median [IQR]		24	127 [120–140]	132 [120–140] (n = 12)	120 [120–130] (n = 12)	0.875
	DBP (mmHg)—median [IQR]		24	80 [70–80]	80 [71.5–80] (n = 12)	80 [70–82.5] (n = 12)	0.200
	Heart rate (bpm)—mean ± SD		23	67.2 ± 10.3	65.8 ± 9.9 (n = 11)	68.4 ± 10.9 (n = 12)	0.558
	Serum creatinine (mg/dL)—mean ± SD		30	1.06 ± 0.34	1.01 ± 0.37 (*n* = 14)	1.08 ± 0.32 (*n* = 16)	0.691
Echocardiographic data	LV EF (Teicholz, %)—mean ± SD		29	64.6 ± 10.9	68.5 ± 6.5 (*n* = 14)	60.9 ± 13 (*n* = 15)	0.061
	S_Diam_, LV (mm)—mean ± SD		29	32.5 ± 6.2	30.5 ± 5 (*n* = 14)	34.3 ± 6.8 (*n* = 15)	0.097
	D_Diam_, LV (mm)—mean ± SD		29	51.2 ± 6.8	50.7 ± 7.8 (*n* = 14)	51.6 ± 6.1 (*n* = 15)	0.734
	Septum (mm)—mean ± SD		28	10.1 ± 1.6	9.6 ± 1.5 (*n* = 14)	10.6 ± 1.5 (*n* = 14)	0.120
	Posterior wall (mm)—mean ± SD		28	9.4 ± 1.2	9.5 ± 1.2 (*n* = 14)	9.3 ± 1.3 (*n* = 14)	0.653
	Aorta (mm)—median [IQR]		28	42 [35.7–49]	42.5 [41.2–48] (*n* = 14)	36.5 [32.2–49] (*n* = 14)	0.112
	Left atrium (mm)—mean ± SD		28	35.9 ± 6.8	34 ± 8.1 (*n* = 14)	37.9 ± 4.7 (*n* = 14)	0.136
	Aortic regurgitation	Absent or mild—*n* (%)	29	18 (62)	11 (37.9)	7 (24.1)	0.072
		Moderate—*n* (%)		7 (24.1)	3 (10.3)	4 (13.8)	
		Severe—*n* (%)		4 (13.8)	0 (0)	4 (13.8)	
	Aortic stenosis	Absent or mild—*n* (%)	29	27 (93.1)	13 (44.7)	14 (48.3)	0.861
		Severe—*n* (%)		2 (6.9)	1 (3.4)	1 (3.4)	

Abbreviations: AAoA: ascending aortic aneurysm; bpm: beats per minute; CAD: coronary atherosclerotic disease; COPD: chronic obstructive pulmonary disease; CTA: computed tomographic angiography; DBP: diastolic blood pressure; D_Diam_ diastolic diameter; EF: ejection fraction; IQR: interquartile range; LV: left ventricle; NYHA: New York Heart Association; SBP: systolic blood pressure; SD: standard deviation; S_Diam_: systolic diameter. *p*: probability of Type I error. All *p* values were > 0.05. *p* values ≤ 0.05 were considered to indicate statistical significance.

**Table 2:** Ascending aortic volume and maximum diameter.

Volume of the Ascending Aorta	Values (30 Patients)	Growth		*p* Value
		No (14 Patients)	Yes (16 Patients)	
Volume first CTA (cm^3^)—median [IQR]	143.80 [126.67–168.18]	141.90 [127.38–164.75]	149.28 [127.82–168.80]	0.324
Volume second CTA (cm^3^)—median [IQR]	149.85 [123.32–176.45]	132.73 [119.63–164.03]	168.71 [144.35–191.12]	0.006
Percentile volume variation between the first and second CTA—mean ± SD	4.53 ± 10.40	[(−4.83) ± (5.86)]	12.71 ± 5.20	0.004
Maximum diameter at first CTA (cm)—mean ± SD	50.41 ± 3.49	49.73 ± 3.89	51.01 ± 3.10	-
Maximum diameter at second CTA (cm)—mean ± SD	52.36 ± 5.17	49.71 ± 4.45	54.68 ± 4.71	-
Diameter variation between the first and second CTA—mean ± SD	1.65 [0.00–3.57]	0.50 [(−1.00)–(1.22)]	3.38 [1.50–5.00]	-

Abbreviations: CTA: computed tomographic angiography; IQR: interquartile range; SD: standard deviation. *p* values ≤ 0.05 were considered significant.

**Table 3:** Wall pressure and wall shear stress corresponding to the first CTA exams and their statistical correlation independently associated with AAoA growth.

Variables		Stress (Pa)	Total (*n*)	First CTA Mean ± Standard Deviation	Growth		*p* Value
					No	Yes	
Pressure ≥100 Pa	Mean	$\bar{P_{100}}$	30	174.4 ± 51.4	154.1 ± 29.5 (*n* = 14)	192.2 ± 60.3 (*n* = 16)	0.041
	Maximum	$P_{max}$	30	321.0 ± 181.2	249.6 ± 100.5 (*n* = 14)	383.4 ± 214.0 (*n* = 16)	0.041
Shear stress ≥5 Pa and ≥7 Pa	Mean	$\bar{{\tau_{s}}_{5}}$	30	7.1 ± 1.1	6.7 ± 0.7 (*n* = 14)	7.4 ± 1.3 (*n* = 16)	0.093
		$\bar{{\tau_{s}}_{7}}$	30	8.9 ± 1.0	8.6 ± 0.7 (*n* = 14)	9.1 ± 1.1 (*n* = 16)	0.109
	Maximum	${\tau_{s}}_{max,5}$	30	18.5 ± 6.6	17.5 ± 6 (*n* = 14)	19.5 ± 7.1 (*n* = 16)	0.415
		${\tau_{s}}_{max,7}$	30	18.5 ± 6.6	17.5 ± 6 (*n* = 14)	19.5 ± 7.1 (*n* = 16)	0.415
Intersection region	Mean value at intersection	$\bar{{\tau_{s}}_{5-100}}$	26	7 ± 1.3	6.5 ± 0.7 (*n* = 11)	7.4 ± 1.4 (*n* = 15)	0.063
		$\bar{{\tau_{s}}_{7-100}}$	26	8.7 ± 1.1	8.2 ± 0.8 (*n* = 12)	9.2 ± 1.2 (*n* = 14)	0.023
	Maximum value at intersection	${\tau_{s}}_{max,5-100}$	27	15.8 ± 6.0	13.7 ± 4.5 (*n* = 12)	17.5 ± 6.6 (*n* = 15)	0.098
		${\tau_{s}}_{max,7-100}$	26	16.2 ± 5.9	13.6 ± 4.6 (*n* = 12)	18.3 ± 6.2 (*n* = 14)	0.041

Abbreviations: CTA: computed tomographic angiography; AAoA: ascending aortic aneurysm; $\bar{P}$: mean pressure in the high-pressure area (i.e., area with pressure ≥100 Pa); $P_{max}$: maximum pressure in the high-pressure area (≥100 Pa); $\bar{{\tau_{s}}_{5}}\;$and $\bar{{\tau_{s}}_{7}}$: mean shear stress in the wall of the area of high shear stress (≥5 Pa and ≥7 Pa, respectively); ${\tau_{s}}_{max,5}$, and ${\tau_{s}}_{max,7}$: maximum wall shear stress in the area of high stress (≥5 Pa and ≥7 Pa, respectively); $\bar{{\tau_{s}}_{5-100}}\;$and $\bar{{\tau_{s}}_{7-100}}$[Pa]: mean wall shear stress in the area of intersection between the areas of high pressure (≥ 100 Pa) and high shear stress (≥5 Pa and ≥7 Pa, respectively); ${\tau_{s}}_{max,5-100}\;{\tau_{s}}_{max,7-100}$: maximum wall shear stress in the area of intersection between the areas of high pressure (≥100 Pa) and high shear stress (≥5 Pa and ≥7 Pa, respectively). *p* values ≤ 0.05 were considered significant.

**Table 4:** Annualized rated of independently significant wall pressure and wall shear stress and their statistical correlation with AAoA growth (univariate logistic regression model).

Variables from the First CTA × Δt	OR (95% CI)	*p* Value
$\bar{P_{100}}\;$	1.006 (CI: 1.000–1.013)	0.057
$P_{max}\;$	1.003 (CI: 1.000–1.006)	0.042
$\bar{{\tau_{s}}_{7-100}}$	1.090 (CI: 0.946–1.250)	0.241
${\tau_{s}}_{max,7-100}$	1.050 (CI: 0.985–1.110)	0.138

Abbreviations: AAoA: ascending aortic aneurysm; CTA: computed tomographic angiography; Δt: time between the first and the second CTA, in years; $\bar{P}$: mean pressure in the high-pressure area (i.e., area with pressure ≥100 Pa); $P_{max}$: maximum pressure in the high-pressure area; $\bar{{\tau_{s}}_{7-100}}$[Pa]: mean wall shear stress in the area of intersection between the areas of high pressure and high shear stress (≥7 Pa); ${\tau_{s}}_{max,7-100}$: maximum wall shear stress in the area of intersection between the areas of high pressure and high shear stress. OR: odds ratio, CI: confidence interval. *p*: probability of Type I error. *p* values ≤ 0.05 were considered significant.

## Data Availability

The data used and analyzed in this study will be made available upon fair request to the corresponding author.
